# Shifting Paradigms in Bronchopulmonary Dysplasia: From Treatment to Etiology/Pathophysiology-Based Classification

**DOI:** 10.3390/biomedicines13040985

**Published:** 2025-04-17

**Authors:** Fumihiko Namba, Hidehiko Nakanishi

**Affiliations:** 1 Department of Pediatrics, Saitama Medical Center, Saitama Medical University, Kawagoe 350-8550, Saitama, Japan; 2 Research and Development Center for New Medical Frontiers, Department of Advanced Medicine, Division of Neonatal Intensive Care Medicine, Kitasato University School of Medicine, Sagamihara 252-0374, Kanagawa, Japan; n0n.hide@med.kitasato-u.ac.jp

**Keywords:** bronchopulmonary dysplasia, extremely low-birth-weight infants, placenta, small for gestational age

## Abstract

Bronchopulmonary dysplasia (BPD) is a severe chronic respiratory disease linked to preterm births. A scoping review was performed to identify risk factors for moderate and severe BPD to develop an evidence-based, early prognostic, globally recognized, and etiology/pathophysiology-based classification. The findings were then validated against a Japanese national database, the Neonatal Research Network Japan. After identifying histological chorioamnionitis, bubbly/cystic appearance on chest X-ray, and small-for-gestational-age infants as risk factors for severe BPD, BPD was divided into nine categories based on the presence or absence of these three risk factors. After consensus was reached using the Delphi method, public comments were requested, and the classification of BPD was finalized. This perspective introduces the new etiology/pathophysiology-based BPD classification, which should be used in research to better understand the respiratory prognosis and pathophysiology of BPD.

## 1. Introduction

Bronchopulmonary dysplasia (BPD) is a chronic respiratory disease linked to preterm birth. It is a serious respiratory complication that frequently occurs, particularly in extremely preterm infants born before 28 weeks gestation. The pathogenesis of BPD has shifted from oxygen and ventilator-induced lung injury (old BPD) first described by Northway et al. in 1967 [1] to arrested or delayed alveolar development (new BPD) in more premature infants, as disclosed by Jobe in 1999 [2].

The definition and classification of BPD have evolved over the last 50 years, with treatment-based approaches, such as oxygen administration, concentration, and the mode and flow rate of respiratory support [3,4,5,6,7,8,9]. This approach has several drawbacks. First, the diagnosis and severity may differ depending on the treating physician. Second, it merely represents short-term findings, rendering it impossible to intervene early for each patient in different conditions and predict long-term respiratory prognosis. In Japan, however, a classification of BPD based on etiology and pathophysiology has used for the past 30 years [10,11], with efforts being made to provide detailed, tailored treatments for each type of BPD.

Since 2021, we have been conducting BPD research as part of the Rare/Intractable Disease Research Project of the Ministry of Health, Labor and Welfare (MHLW) (Title: Research on the Development of Diagnostic Criteria and Classification of Bronchopulmonary Dysplasia, Establishment of Disease Registry, and Preparation of Medical Practice Guidelines; PI: Fumihiko Namba). This viewpoint proposes a modified etiology/pathophysiology-based classification of BPD.

## 2. The Process of Creating the Old BPD Classification and Its Problems

The diagnostic criteria and disease classification for BPD developed by a research group of the Ministry of Health and Welfare (MHW) about 30 years ago (Ogawa 1992, Fujimura 1996) have been utilized in Japan for the last three decades [10,11]. The criteria defined BPD as “respiratory distress symptoms that require oxygen administration as a result of lung abnormalities, excluding congenital malformations, starting in the neonatal period and extending beyond 28 days of age”. The BPD patients were divided into seven categories based on the existence or lack of respiratory distress syndrome (RDS), intrauterine infection/inflammation, including high umbilical cord blood immunoglobulin M (IgM) levels, chorioamnionitis, and funisitis, and diffuse bubbly/cystic appearances on chest X-rays (Supplementary Figure S1) [10,11]. In a 2005 national survey of BPD, Minami et al. found that types I, III, and IV with a diffuse bubbly/cystic appearance on chest X-ray were associated with greater incidence of oxygen and ventilation use at 36 weeks postmenstrual age (PMA) and home oxygen therapy, longer duration of oxygen and ventilator use, and neonatal intensive care unit hospitalization than those without such appearances. This classification of BPD was established between 1989 and 1991 as part of a collaborative research project of the Psychosomatic Disorders Research Group of the Ministry of Health and Welfare (MHW), “Comprehensive Study on Life Management in Neonates and Infants” (PI: Yunosuke Ogawa), which aimed to study BPD. Ogawa et al. found that diagnostic criteria and disease classifications were required for a nationwide survey of BPD, and they examined disease classification criteria based on previous research findings. At the time, Fujimura et al. stated that infants with Wilson–Mikity syndrome, a chronic lung disease in newborns, did not suffer from RDS and had an elevated level of evidence of intrauterine infection/inflammation, including chorioamnionitis, subacute necrotizing funisitis, and high IgM levels [12]. Furthermore, they described 10 cases of chronic respiratory insufficiency with elevated IgM levels and a bubbly/cystic appearance on chest X-rays [13]. Based on these outcomes and additional studies, three factors were determined as critical for the classification of BPD: (1) RDS, (2) intrauterine infection/inflammation, and (3) a bubbly/cystic appearance on chest X-ray.

Given that the BPD classification was first developed 30 years ago, in recent years, it has become more common to hold workshops to discuss the possibility that the classification is obsolete and needs to be revised. Ito and colleagues conducted a cross-sectional questionnaire survey on the diagnosis and classification of BPD at 107 tertiary perinatal centers across the country and published their findings, which had a response rate of 60% [14]. According to the report, 82% of the centers revealed that many BPD cases could not be readily distinguished using the current BPD classification, while only 27% of the centers suggested using the Japanese classification in the future. Furthermore, negative opinions toward the BPD classification were also scattered, indicating that the current BPD classification did not correspond well with the clinical situation. As a result, the authors determined that a consensus on BPD classification may be worth reconsidering [14].

## 3. Creating a New BPD Classification

As previously indicated, the former BPD classification, which has been employed in Japan for the past 30 years, has given rise to several concerns. These include the challenges associated with the objective assessment of BPD classification, the restriction of its application to a single nation, Japan, and the variation in the timing and methodology of evaluating chest X-ray images for the purpose of BPD classification [14]. Therefore, the goal of this study was to create a novel BPD classification system that is (1) evidence-based, (2) predicts early prognosis, (3) is practical not only in Japan but also in other countries, and (4) is based on etiology and pathophysiology. This project was carried out as part of the Research on Measures for Intractable Diseases, Ministry of Health, Labor and Welfare of Japan (MHLW, PI: Fumihiko Namba).

### 3.1. Identifying the Risk Factors for Moderate and Severe BPD

First, to decide which factors should be included in the new BPD classification, a thorough scoping review of the published literature was conducted to identify risk factors for severe BPD as an outcome. A scoping review is a type of review that falls somewhere between a narrative review and a systematic review, such as a Cochrane review, but does not include a research quality assessment or meta-analysis. A scoping review is a useful tool for quickly summarizing the key concepts, information sources, available literature within a research area, and the type of information or evidence [15]. Given these advantages, we chose a scoping review as the best approach for identifying potential risk factors linked to the onset and severity of BPD and respiratory outcomes in this project. First, the population, concept, and context were defined as follows, and the literature was screened using the online databases PubMed and Ichushi, which contain articles published in Japanese-language medical journals. The population being studied consists of extremely preterm infants born at less than 28 weeks of gestation. The concept spans the years 2002 to 2021, during which the studies were carried out in developed countries and written in English or Japanese. The studies included randomized controlled trials, cohort studies, and case–control studies, with more than 500 eligible cases and in which the endpoint was severe BPD classified by the NIH 2001 definition [7]. Descriptive research design studies, animal model, in vitro studies, and studies that evaluated congenital airway diseases such as diaphragmatic hernia and congenital pulmonary airway malformations were excluded. Two independent researchers used the Rayyan software to screen the literature. The extracted data included the risk factors for severe BPD, as well as their respective risk/odds ratios (ORs), 95% confidence intervals (CIs), and P values from the selected literature [16]. A total of 7954 English and 235 Japanese articles were initially screened to identify their titles, abstracts, and content. Ultimately, 3 articles investigated the risk factors for severe BPD, while 32 articles examined looked at factors for mild or moderate BPD. The risk factors for moderate or severe BPD were determined to include small-for-gestational age (SGA) infants (10 articles reported that risk was linked to BPD onset/10 articles reviewed), male sex (4/4), chorioamnionitis (3/3), preterm premature rupture of membranes (2/2), resuscitation (2/2), and a bubbly cystic appearance on X-ray (1/1) [17].

### 3.2. Validation of the Risk Factors

The risk factors for moderate or severe BPD discovered in a thorough literature search were determined using data from a Japanese database (Neonatal Research Network Japan, NRNJ), which included about 65% of very-low-birth-weight or preterm infants born at less than 32 weeks of gestation. The study included 15,834 extremely preterm infants from the NRNJ. BPD severity was classified using the NIH 2001 classification [7]. The study comprised 3783 (24%) infants with no BPD, 5548 (35%) with mild BPD, 4781 (30%) with moderate BPD, and 1722 (11%) with severe BPD. A multivariate analysis found that perinatal factors were significantly linked to severe BPD. These included gestational age [adjusted OR (aOR) 0.83 (95% CI 0.79–0.86)], chorioamnionitis [aOR 1.20 (95% CI 1.06–1.36)], SGA [aOR 1.73 (95% CI 1.51–1.98)], and bubbly/cystic appearance on chest X-ray [aOR 1.79 (95% CI 1.60–2.01)] [18]. Finally, the detection and verification of risk factors for severe BPD using the scoping review and the NRNJ database resulted in the adoption of chorioamnionitis, bubbly/cystic appearance on chest X-ray, and SGA as classification items for the new BPD classification.

### 3.3. Development and Consensus of the Proposed BPD Classification

**Figure 1:**
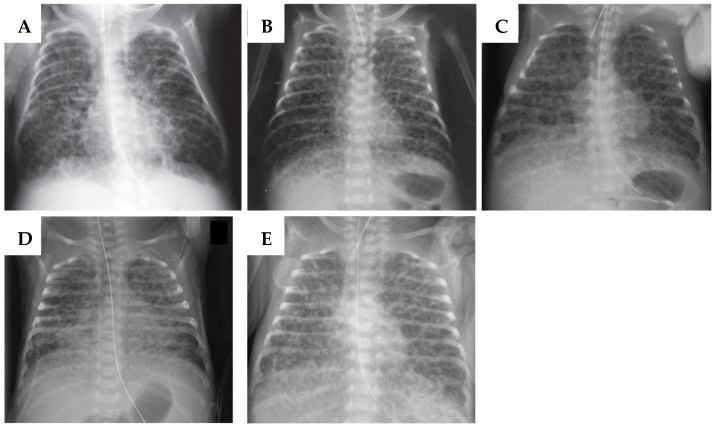
Reference X-rays for the appearance of bubbly/cystic tissue. The X-ray data used in this BPD classification came from the following publications: (**A**) Arai H. *Acta Paediatr*. 2020 [22]; (**B**) Namba F. *Pediatr Int*. 2016 [26]; (**C**) Hirata K. *Arch Dis Child*. 2015 [23]; (**D**) Muehlbacher T. *Children*. 2021 [25]; (**E**) Morris S. *Current Paediatrics*. 2003 [24].

Chorioamnionitis, a bubbly/cystic appearance on chest X-ray, and SGA were used as criteria for the classification (Table 1). SGA infants are those weighing less than the 10th percentile [19]. SGA infants were assigned an “s” alongside the type of BPD, e.g., I (s), II (s), III (s), and IV (s). Chorioamnionitis is based on histological findings, regardless of the stage described by Blanc [20] or Redline [21]. Chest X-ray changes were examined before or at 28 days of age. The diffuse bubbly/cystic appearance is characterized as at least 75% of all lung fields containing bubbles or cysts with a diameter of 1.0–10.0 mm, as seen on chest X-rays (Figure 1) [22,23,24,25,26]. The absence of an objective indicator for determining the presence of bubbly/cystic appearance on chest X-ray necessitates the utilization of the reference provided in Figure 1 as a guide. Without pathological findings, the patient was deemed as type V (unclassifiable), regardless of whether chest X-ray findings were present or absent.

Following the presentation of the recommended new BPD classification and the formation of consensus among experts in various fields using the Delphi method [27,28,29], public comments were solicited through the website of the Japanese Society for Neonatal Health and Development and other related academic societies. Responses to public comments were made accessible for review, and the new BPD classification was determined.

### 3.4. Prognostic Ability of the New BPD Classification

To examine the theory that the new BPD classification can accurately predict long-term respiratory outcomes in BPD patients, Nakanishi and colleagues performed a retrospective multicenter cohort study. The study comprised 15,834 infants delivered at <28 weeks’ gestational age at NRNJ from 2003 to 2016. Of these, 1722 (11%) were classified as having severe BPD (NICHD 2001). Of the 7061 children followed for at least 3 years, 250 (3.5%) required home oxygen therapy (HOT). Histological chorioamnionitis, SGA, and bubbly/cystic appearance on postnatal day 28 were all significantly linked to HOT at 3 years of age [aOR 1.54 (95% CI 1.14–2.08)], [aOR 1.70 (95% CI 1.21–2.39)], and [aOR 2.63 (95% CI 1.94–3.56)], respectively). In comparison to infants without these three risk factors (type II BPD), infants who had multiple of these factors, particularly in combination with a bubbly/cystic appearance, had greater aORs for receiving HOT at 3 years of age (type I [aOR 2.01 (95% CI 1.05–3.82)]; type I(s) [aOR 4.70 (95% CI 2.36–9.38)]; type III [aOR 3.72 (95% CI 2.11–6.56)]; type III(s) [aOR 5.39 (95%CI 2.40–12.10)]; type VI(s) [aOR 2.60 (95%CI 1.02–6.58)]) (Supplementary Figure S2) [18]. Therefore, the new BPD classification can be used not only to guide intervention selection for each risk factor but also to predict long-term pulmonary outcomes.

## 4. Conclusions

An innovative classification for BPD was established as part of the Research on Measures for Intractable Diseases project, which is sponsored by the Ministry of Health and Welfare of Japan. It is expected that research will be carried out in Japan and other countries to better understand the long-term pulmonary results and pathophysiology of each BPD type.

## Figures and Tables

**Table 1:** New BPD classification by the MHLW research group (2023).

Type ^(a)^	Histological CAM ^(c)^	Bubbly/Cystic Appearance on Chest X-Ray ^(d)^
I (s) ^(b)^	−	+
II (s) ^(b)^	−	−
III (s) ^(b)^	+	+
IV (s) ^(b)^	+	−
V	Unclassifiable ^(e)^	

(a) BPD is classified into five categories based on three criteria: histological CAM, bubbly/cystic appearance on frontal chest X-ray, and SGA. The type of BPD is shown. (b) SGA is categorized as birth weight below the 10th percentile, and “s” is included alongside the type of BPD in the case of SGA (the BPD types without SGA are designated I, II, III, IV, and those with SGA are designated Is, IIs, IIIs, IVs). (c) CAM should be diagnosed histologically, its stage notwithstanding. (d) X-ray changes are evaluated within 28 days of age, and the lungs are split into four areas, first at the level of the right and left lungs, then at the level of the upper and lower sections within each lung. The diffuse bubbly/cystic appearance is characterized as the presence of bubbles or cysts with a diameter ranging from 1.0 to 10.0 mm in three of four areas. (e) Placental histology is advised; however, if histological findings are unknown, the case is classified as Type V, regardless of chest X-ray results. BPD—bronchopulmonary dysplasia; CAM—chorioamnionitis; SGA—small for gestational age.

## Data Availability

The datasets generated during and/or analyzed during the current study are available from the corresponding author on reasonable request.

## Supplementary Materials

The following supporting information can be downloaded at https://www.mdpi.com/article/10.3390/biomedicines13040985/s1, Figure S1: Old BPD classification by the MHW research group (1992, 1996); Figure S2: Associations of each type of BPD with HOT at 3 years of age.

## Abbreviations

The following abbreviations are used in this manuscript:

BPD	Bronchopulmonary dysplasia.
MHLW	Ministry of Health, Labor and Welfare.
MHW	Ministry of Health and Welfare.
RDS	Respiratory distress syndrome.
IgM	Immunoglobulin M.
PMA	Postmenstrual age.
OR	Odds ratio.
CI	Confidence interval.
SGA	Small for gestational age.
NRNJ	Neonatal Research Network Japan.
aOR	Adjusted odds ratio.
HOT	Home oxygen therapy.
