# Supplementary material for: Shifting Paradigms in Bronchopulmonary Dysplasia: From Treatment to Etiology/Pathophysiology-Based Classification
Source: Biomedicines. 2025 Apr 17;13(4):985. doi: 10.3390/biomedicines13040985 (PMC12025045; doi:10.3390/biomedicines13040985)
Supplement: Supplementary file 1 [file biomedicines-13-00985-s001.zip › biomedicines-3581789-supplementary.pptx]

## Slide 1
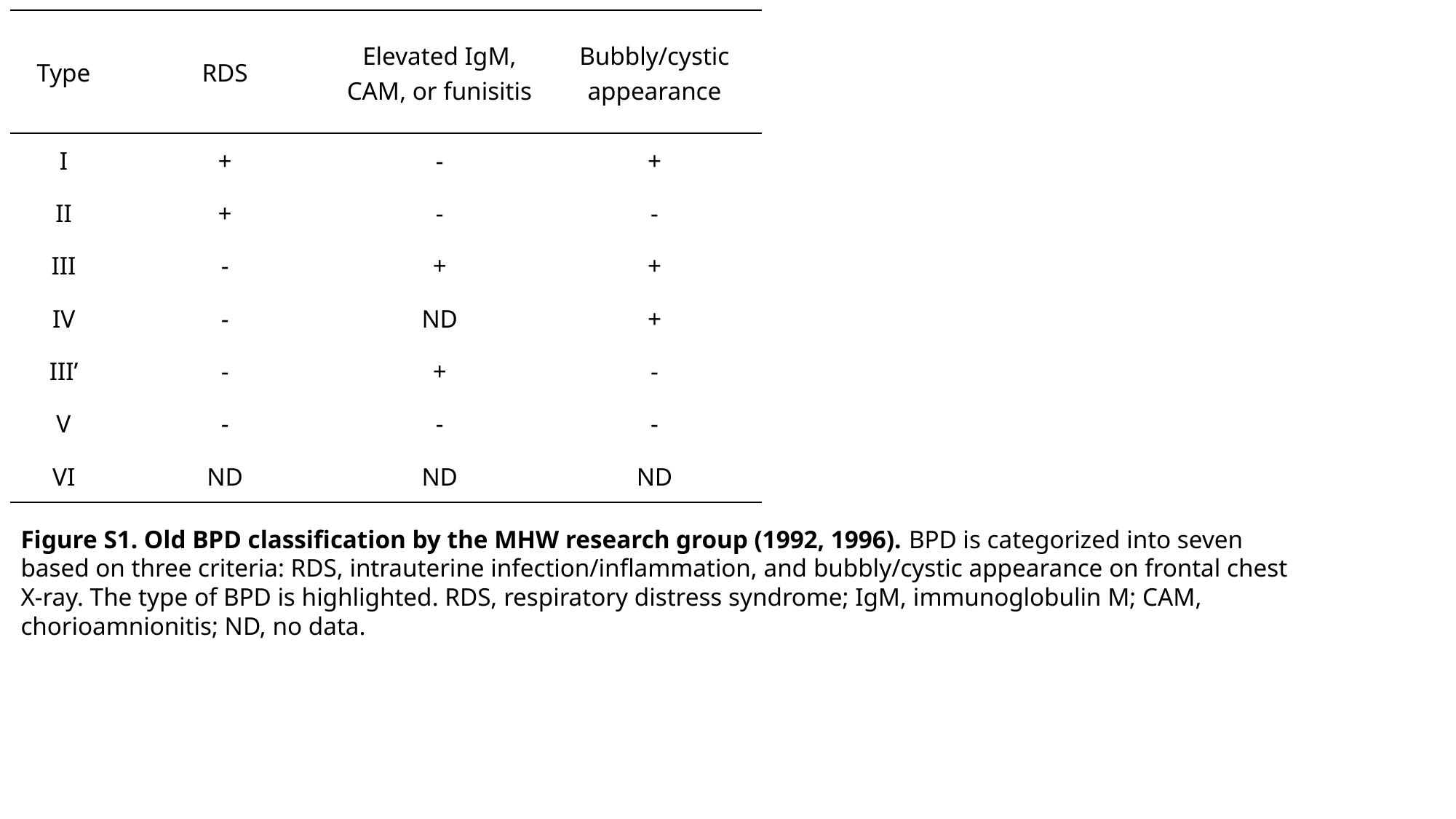

| Type | RDS | Elevated IgM, CAM, or funisitis | Bubbly/cystic appearance |
| --- | --- | --- | --- |
| I | + | - | + |
| II | + | - | - |
| III | - | + | + |
| IV | - | ND | + |
| III’ | - | + | - |
| V | - | - | - |
| VI | ND | ND | ND |
Figure S1. Old BPD classification by the MHW research group (1992, 1996). BPD is categorized into seven based on three criteria: RDS, intrauterine infection/inflammation, and bubbly/cystic appearance on frontal chest X-ray. The type of BPD is highlighted. RDS, respiratory distress syndrome; IgM, immunoglobulin M; CAM, chorioamnionitis; ND, no data.

## Slide 2
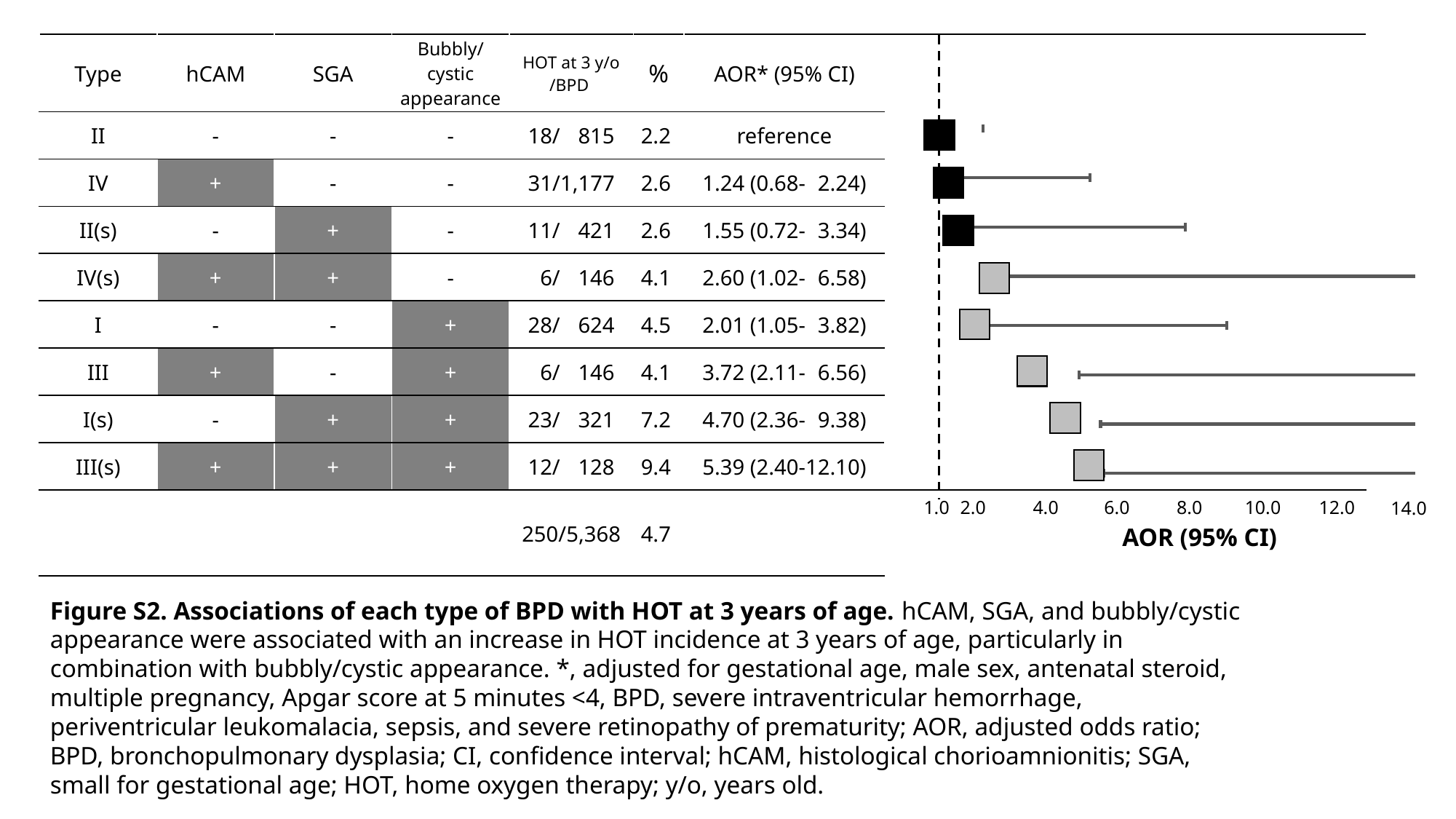

| Type | hCAM | SGA | Bubbly/ cystic appearance | HOT at 3 y/o /BPD | % | AOR\* (95% CI) | |
| --- | --- | --- | --- | --- | --- | --- | --- |
| II | - | - | - | 18/0,815 | 2.2 | reference | |
| IV | + | - | - | 31/1,177 | 2.6 | 1.24 (0.68-02.24) | |
| II(s) | - | + | - | 11/0,421 | 2.6 | 1.55 (0.72-03.34) | |
| IV(s) | + | + | - | 06/0,146 | 4.1 | 2.60 (1.02-06.58) | |
| I | - | - | + | 28/0,624 | 4.5 | 2.01 (1.05-03.82) | |
| III | + | - | + | 06/0,146 | 4.1 | 3.72 (2.11-06.56) | |
| I(s) | - | + | + | 23/0,321 | 7.2 | 4.70 (2.36-09.38) | |
| III(s) | + | + | + | 12/0,128 | 9.4 | 5.39 (2.40-12.10) | |
| | | | | 250/5,368 | 4.7 | | |
### Chart
| Category | |
|---|---|
1.0
2.0
4.0
6.0
8.0
10.0
12.0
14.0
AOR (95% CI)
Figure S2. Associations of each type of BPD with HOT at 3 years of age. hCAM, SGA, and bubbly/cystic appearance were associated with an increase in HOT incidence at 3 years of age, particularly in combination with bubbly/cystic appearance. *, adjusted for gestational age, male sex, antenatal steroid, multiple pregnancy, Apgar score at 5 minutes <4, BPD, severe intraventricular hemorrhage, periventricular leukomalacia, sepsis, and severe retinopathy of prematurity; AOR, adjusted odds ratio; BPD, bronchopulmonary dysplasia; CI, confidence interval; hCAM, histological chorioamnionitis; SGA, small for gestational age; HOT, home oxygen therapy; y/o, years old.
